# Debunking Cannabidiol as a Treatment for COVID-19: Time for the FDA to Adopt a Focused Deterrence Model?

**DOI:** 10.7759/cureus.8671

**Published:** 2020-06-17

**Authors:** Chelsea L Shover, Keith Humphreys

**Affiliations:** 1 Psychiatry and Behavioral Sciences, Stanford University School of Medicine, Palo Alto, USA

**Keywords:** cannabidiol, health policy, food and drug administration, cannabis, covid-19, regulation

## Abstract

Many cannabidiol (CBD) retailers make unsupported medical claims about their product. In recent years, the U.S. Food and Drug Administration (FDA) has sent warning letters to CBD retailers who promoted CBD to treat Alzheimer’s disease, cancer, diabetes, and other serious conditions for which there is no evidence of its efficacy as a treatment or preventive. Compliance with these warning letters has been low. During the novel coronavirus disease 2019 (COVID-19) pandemic, the FDA has begun sending more strongly worded warning letters that appear to have better compliance in that most of these companies have removed COVID-19-related claims. However, many continue to present other unsupported medical claims on other serious medical conditions like cancer, depression, addiction, and bone fractures, among many others. We argue that adopting a strategy of focused deterrence where the FDA prioritizes enforcement related to COVID-19 claims - but when COVID-19-related claims are found, pursues all other violations by that company - would present an opportunity to efficiently cut down on harmful claims overstating CBD’s benefits.

## Editorial

Over the past few years, the U.S. Food and Drug Administration (FDA) has sent warning letters to some cannabidiol (CBD) retailers instructing them to cease making unsubstantiated therapeutic claims regarding many diseases, including cancer, addiction, and diabetes [1]. These letters asked recipients to respond in writing within 15 days and appear to have been largely ignored, as CBD retailers continue making unsupported claims about their product’s ability to treat or prevent a variety of serious conditions. In March, the FDA began responding to unsupported therapeutic claims about CBD and novel coronavirus disease 2019 (COVID-19) by sending more strongly worded warning letters that instruct recipients to withdraw such claims within 48 hours [1]. The FDA has limited regulatory capacity to police the numerous CBD retailers and the even larger number of unsupported therapeutic claims about CBD and other cannabis-derived products, so it is worth considering whether this is an efficient, high-impact strategy [2,3].

To examine whether COVID-19-related letters led to faster and more complete compliance, on April 30, 2020, we reviewed the websites and social media of all CBD retailers who received an FDA warning letter in 2019 or 2020. We expected that a greater proportion of CBD manufacturers that received the strongly worded COVID-19-related warning letters would have removed unsupported medical claims compared to CBD manufacturers that received the letters regarding other unsubstantiated therapeutic claims. We compared the proportion of companies that removed claims or added prominent disclaimers (i.e., positioned near the product and stating that the FDA had not evaluated the claims and products were not intended to diagnose, prevent, or treat disease) by type of warning letter.

In 2019 and 2020, the FDA sent 18 letters to companies about “Unapproved New Drugs/Misbranded/Cannabidiol (CBD) Products.” The recipients included manufacturers and retailers, with many companies doing both. Notably, at least one of the recipients (Curaleaf, Wakefield, MA) operates cannabis dispensaries around the United States in addition to selling CBD online via Curaleaf Hemp, which was cited in the 2019 warning letter. As of April 30, 2020, half (nine) of these companies still marketed CBD as treating or preventing disease without disclaimers. Between March 6, 2020, and April 27, 2020, the FDA sent COVID-19-related warning letters related to 38 companies, 21% (eight) of which had CBD products for sale on their website. As of April 30, 2020, six of the eight companies had either removed the COVID-19-related statements or added prominent disclaimers on the same page as the statements.

However, half of these companies had other medical claims similar to those for which the 2019 companies had been cited (Table 1). Conversely, three of the companies that received letters in 2019 had recently added new content marketing CBD products as protective against COVID-19, or claiming CBD improves immunity generally.

**Table 1:** Online content of CBD retailers that received warning letters from the U.S. Food and Drug Administration CBD, cannabidiol; PTSD, posttraumatic stress disorder; TBI, traumatic brain injury

Company	Citation	Date	COVID-19-related content	Other medical claims (up to one example per company)
Homero Corp DBA Natures CBD Oil Distribution, Manchester, NH	CBD Letter: Unapproved New Drugs/Misbranded/Cannabidiol (CBD) Products	4/20/20		"CBD oil is being discovered to work for numerous health issues, but it is anxiety that it seems to work most effectively. Though medications provided from the pharmaceutical companies and the medical community can be beneficial, there are side effects and the concern for addiction with regular use. There is an array of advantages associated with CBD oil as a treatment for anxiety. Studies show that 1 single dose is efficient and quick and does not have to be used on a daily basis. Other benefits for anxiety sufferers is that if traditional medication fails or cannot be tolerated CBD offers hope for those stuck with no solution. For those who want to add CBD to a traditional medicine to give it a boost, there is no evidence that CBD cannot interact with other drugs and can be used safely."
Nova Botanix LTD DBA CanaBD, London	COVID-19 Letter: Unapproved Products Related to the Coronavirus Disease 2019 (COVID-19)	4/16/20	"Current situation has been striking fear and anxiety amongst many as it goes on a rampage around the world. CBD works with your body to regulate optimal functionality of the receptors that are connected to anxiety, stress and sleep disorders. In addition, CBD may strengthens [sic] your immune system by promoting an overall healthier wellbeing."	"In addition, CBD is known to treat these conditions: joint pain, arthritis, autoimmune disorder, diabetes, high blood pressure, depression, migraines, Crohn’s and colitis, inflammation, nausea, Parkinson’s, rheumatism, skin conditions."
Gaia Arise Farms Apothecary, Waynesville, NC	COVID-19 Letter	4/13/20		"CBD from cannabis improves inflammatory, spastic, and nerve pain, (2) it provides restful sleep, (3) reduction of nightmares for those with PTSD, (4) it reduces anxiety, which causes muscle tension, (5) helps relieve Opioid Addiction, in case the real doctors (or drug dealers) got you hooked. (6) But I’d use our Addiction Protocol, to heal that addiction, along with True Pain Relief w/CBD for the chronic pain."
Earthley, Columbus, OH	COVID-19 Letter	4/9/20	"A LOT of people are worried about coronavirus right now. It doesn’t help that no one seems to know exactly what the truth is. We also don’t know what will happen if or when it dramatically spreads in the US. However, we do NOT think it’s time to panic. But if it helps you to feel like you’re ready, just in case … we’re here. And so we’re giving away an Immune Support Collection to one lucky winner!"	"Experience true relief of back pain, muscle pain, joint pain, and more. Our synergistic cream combines naturally pain-relieving herbs like St. John’s Wort and yarrow with full-spectrum CBD. Rub a small amount into the sore spot and feel better soon!"
CBD Online Store, Laguna Hills, CA	COVID-19 Letter	4/7/20	"Ylan-Ylan is a well-known superfood that boost and support the immune system [sic]. Adding CBD, nature’s strongest anti-inflammatory, will keep your body from becoming inflamed when it throws all it can at a disease, decreasing your risks even more."	"Study: #CBD Helps Heal #Bone #Fractures. What a miraculous molecule CBD is!"
Indigo Naturals, Seattle, WA	COVID-19 Letter	4/6/20	From blog post, "Can CBD help with viral cytokine storm?": "In inflammatory states (such as cytokine storm), CBD can calm inflammation." (Post is tagged: Can CBD help with the cytokine storm response; Covid19 or coronavirus and cytokine storms; the endocannabinoids system and immune response to coronavirus.)	"Here's the running list of CBD benefits: CBD benefits for Addiction; CBD benefits for Allergies and Histamines; CBD benefits for Anxiety (#1!); CBD benefits for Arthritis; CBD benefits for Autoimmune Disease and the Immune System; CBD benefits for Blood Pressure; CBD benefits for Cancer (#5); CBD benefits for Dementia and Alzheimer's; CBD benefits for Depression; CBD benefits for Diabetes."
Native Roots Hemp, Algoma, WI	COVID-19 Letter	4/6/20		"Studies show CBD oil helps with: addiction, anxious feelings, appetite, behavioral issues, blood circulation, blood sugar regulation, bone health, cardio health, cellular health, circulatory health, compulsiveness, digestive health, endocrine health, eye health, focus, inflammation, joint discomfort, kidney function, liver function, lymphatic health, lung health, memory loss, mood stability, motion sickness, muscle degeneration, nervous system health, reproductive health, respiratory health, skin health, sleeplessness, traumatic stress, weight management."
NeuroXPF, Las Vegas, NV	COVID-19 Letter	3/31/20	"Energize your immune system by igniting your endocannabinoid system with CBD! Learn more at http://NeuroXPF.com"	"Research has shown that CBD can improve brain functions and even heal brain tissue damaged by TBIs. Take 15% off your order at http://NeuroXPF.com in honor of #BrainInjuryAwarenessMonth with coupon code: cbd4brain"
Mr. Pink Collections, LLC, Beverly Hills, CA	CBD Letter	11/22/19		"Not only CBD oil can help to relieve health problems such as anxiety, depression, reduce seizures and also combat insomnia but it can also help for muscle recovery."
Whole Leaf Organics, Sherman Oaks, CA	CBD Letter	11/22/19		"CBD-EX combines the best in cancer fighting elements, into one simple capsule." "Our formulations have been proven to be effective at reducing inflammation, and minimizing the way cancer cells manipulate neighbor cells - the key factor in being successful when trying to be proactive against disease."
Infinite Product Company LLLP, Lakewood, CO	CBD Letter	11/22/19	Now selling "Nano CBD Hand Sanitizer"	
Organix Industries/Plant Organix, San Bernardino, CA	CBD Letter	11/22/19		“Vaping CBD oil is one of the preferred methods to ease side effects of diseases, illnesses, and ailments including leukemia, all types of cancers, stress, anxiety, joint pain, seizures, inflammation, sleep apnea, high blood pressure and the list goes on.”
Red Pill Medical, Phoenix, AZ	CBD Letter	11/22/19		"When CBD reaches a family of receptors, or cells that receive stimuli, called vanilloid receptors, the interaction results in lower inflammation and levels and pain perception, according to a study published in Current Neuropharmacology. And a study published in July 2016 in the European Journal of Pain found that CBD could help people with arthritis manage their pain. The animal study looked at whether using a CBD gel transdermally (on the skin) would reduce inflammation and signs of pain, and researchers concluded that the topical product did offer relief from pain-related behaviors without evidence of side effects."
CDRL Nutritional Inc, Concord, CA	CBD Letter	11/22/19		"CBD is a powerful antioxidant that has several beneficial applications to help you with: anxiety, alcoholism, back pain, chronic pain, diabetes, depression, epilepsy, fibromyalgia, migraine, multiple sclerosis, PTSD, psoriasis, rheumatoid arthritis, schizophrenia & psychosis, and much more,"
Private I Salon, Charlotte, NC	CBD Letter	11/22/19	"Although inflammation is often seen in a negative light, it's a vital immune response to infection.... Therefore, preventing inflammation when it is actually needed can lower immune function and make people more susceptible to disease. CBD and other cannabinoids may be able to help balance that function in the body."	"The receptors that CBD oil targets are in the endo-cannabinoid system, which can be found in the nervous system, digestive system, in your brain, and throughout your entire body. When your endo-cannabinoid system is not balanced, you suffer from things like stress, depression, hypertension, fatigue, migraines, chronic pain, and more."
Rooted Apothecary, Naples, FL	CBD Letter	10/10/19	"Save up to 40% - Corona Relief Efforts. No one planned on this.We recently ran a discount and to those of you who purchased product, we continue to be thankful for your support. Now it's our turn to go a step further. We are offering our biggest discount ever! The economic impact is widespread, the fear is more contagious than anything else, and who knows when things will 'normalize.' But, we do know the science behind hemp-derived CBD and how it interacts with the Endocannabinoid System in each one of us. Right now, more than any other time in recent memory, this plant has a BIG chance to prove what it can do."	
Curaleaf, Wakefield, MA	CBD Letter	7/26/19		Claims have been removed from Curaleaf Hemp, which was cited in the FDA letter, but many remain on Curaleaf's other websites and social media accounts, e.g. this from curaleaf.com/az: "You’ve likely heard marijuana referred to, in not so nice terms, as a gateway drug. This long-spanning theory suggests marijuana users are more likely to partake in harder drugs than non-users. Major studies have taken place in the last few years that not only debunked this persistent myth, but have shown the opposite to be true. In fact, the results of recent drug addiction treatment studies show cannabis is effective in helping those addicted to opioids safely withdraw from, or exit, the drug."

Our rapid online review comparing the compliance with these two types of FDA warning letters suggests that the FDA might profitably adopt a “focused deterrence” strategy, which police sometimes use to deter gang violence [4].

A focused deterrence strategy involves publicly telling potential offenders what the number one enforcement priority is (in the police case, typically gun violence; in this case, COVID-related claims) and if anyone breaks the rule, the offender is investigated for all transgressions, even otherwise low-priority ones. A focused deterrence strategy has two effects. First, because the certainty that penalties will occur is more deterrent than a probabilistic threat of even severe punishment, announcing that COVID-19 is the highest priority will prevent other CBD retailers from making such claims in the first place. Second, the marginal cost of expanding enforcement when intervening against CBD claims (i.e., expanding said letters to cover all of that same retailer’s unsupported claims about a range of diseases) is modest, allowing the FDA to completely clean up the worst actors in the industry.

Our review highlighted a few other ways that focused deterrence adopted during a pandemic could have lasting effects even after the acute threat of COVID-19 has diminished. (1) The FDA might consider requiring a response within 48 hours for all misbranded CBD drug products. (2) Several of the companies that received warning letters in 2019 had removed the original offending content but were now selling products aimed at treating or preventing COVID-19 (Table 1). These types of claims and promotions presumably fall under the area of what companies were instructed to avoid in the future. Publicly adopting focused deterrence can reinforce the gains made by earlier enforcement without having to necessarily individually re-contact the CBD companies that have been previously cited. (3) The FDA may consider expanding letters to cover all websites operated by a company it is citing. Curaleaf provides a useful example. Cited in July for its subsidiary Curaleaf Hemp, Curaleaf manufacturers CBD and cannabis products and operates dispensaries in several states. The dispensaries were not mentioned in the warning letter, and despite removing CBD-related medical claims from the cited website, medical claims about cannabis products were still present on Curaleaf’s state websites and social media as of April 30, 2020. Again, it would require only marginal additional resources for FDA to expand the warning to cover all such claims from a retailer, including when they are made on a different subsidiary’s website.

In conclusion, we found that FDA letters regarding non-COVID-focused claims by CBD retailers have made little impact - few letters have been sent and nearly half have been ignored. But the stricter COVID-19 letters were complied with at a higher rate, despite the much shorter time window since they were sent. However, the fact that about half of the retailers that received COVID-19 letters still had other unsupported medical claims on their websites or social media is concerning. This suggests that significant public health benefit at marginal cost could be garnered if FDA letters sent in response to COVID-19 claims were amended to encompass all unsupported therapeutic claims made by recipients. Moreover, making it explicit and public that this focused deterrence strategy was being employed could prevent the industry as a whole from making COVID-19-focused claims in the first place.

## References

[1] (2020). U.S. Food and Drug Administration warning letters. https://www.fda.gov/inspections-compliance-enforcement-and-criminal-investigations/compliance-actions-and-activities/warning-letters.

[2] Allem JP, Escobedo P, Dharmapuri L (2020). Cannabis surveillance with Twitter data: emerging topics and social bots. Am J Public Health.

[3] Caputi TL (2020). The medical marijuana industry and the use of “research as marketing”. Am J Public Health.

[4] Kennedy DM (2009). Deterrence and Crime Prevention (1st Edition), Routledge Studies in Crime and Economics. https://books.google.com/books/about/Deterrence_and_Crime_Prevention.html?id=jhcpAQAAIAAJ.

